# Psychosocial Predictors of Bruxism

**DOI:** 10.1155/2019/2069716

**Published:** 2019-10-13

**Authors:** Agnieszka Przystańska, Aleksandra Jasielska, Michał Ziarko, Małgorzata Pobudek-Radzikowska, Zofia Maciejewska-Szaniec, Agata Prylińska-Czyżewska, Magdalena Wierzbik-Strońska, Małgorzata Gorajska, Agata Czajka-Jakubowska

**Affiliations:** ^1^Department of Temporomandibular Disorders, Division of Prosthodontics, Poznań University of Medical Sciences, Poznań, Poland; ^2^Institute of Psychology, Adam Mickiewicz University in Poznań, Poznań, Poland; ^3^Faculty of Medicine, Katowice School of Technology, Katowice, Poland

## Abstract

**Objectives:**

The study aimed to investigate the psychosocial predictors of bruxism. The association of various psychosocial factors such as alexithymia, emotional processing, state and trait anxiety, and stress with awake bruxism was analysed.

**Methods:**

The study involved 52 volunteers diagnosed with awake bruxism. The toolkit that was used included the Toronto Alexithymia Scale (TAS-20), the Emotional Processing Scale (EPS), the Cohen Perceived Stress Scale (PSS-10), and the State- and Trait-Anxiety Inventory (STAI), with independent individual psychological diagnoses being made for every patient. The results were statistically analysed using IBM SPSS Statistics 24.

**Results:**

The obtained data clearly show that psychological traits—both permanent dispositions (e.g., state anxiety and alexithymia) and temporary states (e.g., trait anxiety, emotional processing deficits, and psychological stress)—are significant determinants of awake bruxism. The percentage of explained variance indicates the presence of other factors as well.

**Conclusions:**

Psychosocial factors such as state anxiety and trait anxiety, alexithymia, and perceived stress are as important as somatic causes in the occurrence and maintenance of awake bruxism. The profile of the obtained data suggests the possibility of preventing or minimizing the symptoms of awake bruxism through properly constructed psychoprophylactic interactions.

## 1. Introduction

Bruxism, a repetitive activity of masticatory muscles characterized by grinding or clenching the teeth, can occur during sleep (sleep bruxism(SB)) or during wakefulness (awake bruxism (AB)). Recently, the definition of bruxism was corrected by Lobezzo et al. [1]: SB is a masticatory muscle activity during sleep (characterised as rhythmic and nonrhythmic); awake bruxism is a masticatory muscle activity during wakefulness (characterised by repetitive or sustained tooth contact and/or by bracing or thrusting the mandible). In otherwise healthy individuals, bruxism should be considered as a behavior that can be a risk factor for certain clinical consequences [2]. The epidemiologic characteristics of bruxism are not clear due to different diagnostic strategies and the investigation of nonrepresentative populations [2, 3]. The prevalence of “sleep bruxism” varies from 9.3% to 15.9% and the prevalence of “awake bruxism” varies from 22.1% to 31% in the adult population [3–9]. The prevalence of generally identified “bruxism” has been reported in 8% to 31.4% of the population [10, 11]. It is believed that the presence of comorbid conditions, such as other psychological diseases, may influence the assessment of bruxism's prevalence [3].

Although widespread among populations and repeatedly investigated, bruxism remains an enigmatic disease with many of its aspects requiring further scientific evaluation [12–14]. Bruxism is considered to be a multifactorial disorder [12, 15–22], and its aetiology is not well defined. Various factors, i.e., tooth interference in dental occlusion [23, 24], sleep arousal episodes [25, 26], central nervous system-associated causes involving neurotransmitters and basal ganglia [15, 27–29], and side imbalances in striatal D2 receptor bindings [29], have been identified as related to bruxism. Some studies suggest the possible involvement of genetic factors in the pathogenesis of bruxism [13, 30, 31]. Various psychosocial factors associated with bruxism have also received much attention in the literature. A number of studies have shown a relationship with bruxism of certain personality traits (e.g., aggression or emotional suppression) [1, 15, 31–34], psychosocial factors (e.g., perceived time pressure or competition) [35, 36], and psychological stress (cf. stressful lifestyle) [8, 35–39]. Anxiety and neuroticism personality traits have especially been reported in individuals with bruxism [1, 38–41]. By inference, bruxism is supposed to be inducted centrally, with the somatic effects found in the stomatognathic system (i.e., muscle tenderness, limitation of jaw movements, oral and facial pain, headache, and tooth wear or fracture) [14]. Regardless of the definition, bruxism, being a somatic symptom disorder, is related in time with stressful events or problems. Nonetheless, the literature does not provide a definite conclusion whether bruxism is associated with psychological dispositions or transient states of a psychosocial character. Therefore, the aim of the present study was to investigate whether various psychosocial factors such as alexithymia, emotional processing deficits, and anxiety and stress correlate with bruxism.

## 2. Materials and Methods

### 2.1. Investigated Group

The subjects of this study were 54 first-visit patients (38 women, 16 men) who visited the Department of Temporomandibular Disorders because of tooth wear and feeling of facial tiredness. The patients underwent routine examination procedures including a bruxism questionnaire and an RDC/TMD questionnaire. Additionally, during examination, the following data were gathered: tooth wear (using Martin's tooth wear index), vertical enamel fractures in the anterior teeth, indentations along the lateral borders of the tongue (tongue scalloping), and maceration of the buccal mucosa at the level of the bite line (linea alba).

Bruxism assessment: both awake and sleep bruxism were examined on the basis of a self-reporting questionnaire. The bruxism questionnaire contained five questions giving 3 possible answers to patients: yes/no/I do not know.

1. Are you aware of the fact that you clench your teeth when awake?
2. Do you have your jaws thrust on morning awaking?
3. Do you grind your teeth when awake?
4. Are you aware of the fact that you grind your teeth when asleep?
5. Has anyone told you that you grind your teeth when asleep?

Afterwards the patients were interviewed and received a clinical examination according to the criteria described by Peasani et al. [42] and taking into consideration the results of Lavinge et al. [43]. The patients were examined by two clinical specialists in temporomandibular disorders working at the Department.

1. Awake clenching: positive history of tooth clenching when awake confirmed by the patient during interview and at least two of the following signs/symptoms: pain during palpation in one masseter muscles site per side (RDC/TMD), hypertrophy of masseters, presence of linea alba, and tongue scalloping
2. Awake grinding: positive history of tooth grinding while awake confirmed by the patient during interview along with the following clinical findings: noticeable tooth wear on the incisal surfaces of the anterior teeth or/and on the guiding cusps of the posterior teeth
3. Sleep clenching: positive history of tooth clenching during sleep confirmed by the patient during interview and at least two of the following signs/symptoms: pain during palpation in one masseter muscles site per side (RDC/TMD), hypertrophy of masseters, presence of linea alba, and tongue scalloping
4. Sleep grinding: positive history of tooth grinding during sleep confirmed by the patient during interview along with the following clinical findings: noticeable tooth wear on the incisal surfaces of the anterior teeth or/and on the guiding cusps of the posterior teeth

This method was used as we considered that the interview along with a clinical assessment of the most probable bruxism-related signs and symptoms may be sufficient to diagnose bruxism.

Finally, 52 patients were diagnosed with awake bruxism and 2 with sleep bruxism.

The psychological assessments only included the 52 people with awake bruxism (36 women, 16 men).

### 2.2. Method

The University Bioethical Committee (Decision 1198/17) approved the study protocol.

The one-stage study took place during the patient's second visit to the clinic, between November 2017 and May 2018, in a specially arranged room in the presence of a psychologist. Prior to participation in the study, the patients expressed written, informed consent. Then, the patients completed a “pencil and paper” research toolkit containing the Toronto Alexithymia Scale (TAS-20), the Emotional Processing Scale (EPS), the Cohen Perceived Stress Scale (PSS-10), and the State- and Trait-Anxiety Inventory (STAI). The mean time for this part of the study was 30 mins. Based on the results obtained, independent individual psychological diagnoses were made for every patient. The diagnosis included the characteristics of the patient's functioning in terms of the traits studied. The diagnoses were made available to the participants by e-mail.

### 2.3. Measurement

1. The Toronto Alexithymia Scale 20 (TAS–20) [44] measures alexithymia. Alexithymia refers to a phenomenon in people who have trouble identifying and describing emotions and who tend to minimize emotional experiences and focus attention externally. The TAS is a 20-item instrument. The scale has three measuring subscales: Difficulty Describing Feelings Subscale (e.g., “I am often puzzled by sensation in my body”); Difficulty Identifying Feeling Subscale (e.g., “It is difficult for me to find the right words for my feelings”); and Externally Oriented Thinking Subscale (e.g., “I find an examination of my feelings useful in solving personal problems (reverse keyed)”). Items are rated using a 5-point Likert scale whereby 1 = strongly disagree and 5 = strongly agree. The total alexithymia score is the sum of responses to all 20 items, while the score for each subscale factor is the sum of the responses to that subscale.
2. The Emotional Processing Scale (EPS) [45–47] is a 25-item questionnaire designed to identify emotional processing styles and potential deficits. The EPS uses five subscales of five items each to generate a total emotional processing score. The subscales are as follows: Suppression (e.g., “I smother my feelings”); Signs of Unprocessed Emotion (e.g., “Unwanted feelings keep intruding”); Controllability of Emotion (e.g., “I react too much to what people say or do”); Avoidance (e.g., “I try to only talk about pleasant things”); and Emotional Experience (e.g., “My emotions feel blunt/dull”). Items were rated using a 10-point Likert scale whereby 0 = completely disagree and 9 = completely agree. The total emotional processing score is the sum of responses to all 25 items, while the score for each subscale factor is the sum of the responses to that subscale. If the total for each subscale sum is high, the deficit is interpreted as more intensive.
3. The Perceived Stress Scale (PSS-10) [48, 49] (PSS-10 is distributed in Poland by the Psychological Test Laboratory of the Polish Psychological Association (https://www.practest.com.pl/npsr-narzędzia-pomiaru-stresu-i-radzenia-sobie-ze-stresem)) measures the perception of stress in the last month. A total of 10 items produced a PSS score. It was possible to indicate two subscales on the basis of factor analysis: perceived helplessness (5 items) and perceived self-efficacy (4 items) [49]. The participant evaluates the items, on a 5-point Likert scale, as to how often the described situations happen, from 0 = “never” to 4 = “very often.”
4. The State-Trait Anxiety Inventory (STAI) [50–52] measures anxiety identified as temporary state connected with a situation and anxiety identified as stable trait connected with personality. The inventory has two parts: X-1 for measuring state anxiety and X-2 for measuring trait anxiety. Each of the parts has 20 items. The participants used a 4-point Likert scale for state anxiety from 1 = “not at all” to 4 = “very much so” and also a 4-point scale for trait anxiety from 1 = “almost never” to 4 = “almost always.”

The results were statistically analysed using IBM SPSS Statistics 24.

## 3. Results and Discussion

The first step used descriptive statistics to analyse the data.  Table 1 shows the maximum and minimum, mean, standard deviation, Cronbach's alpha, and Shapiro–Wilk test's values for all variables. The reliability of the individual tools was acceptable except for the externally oriented thinking subscale in alexithymia. The results obtained in the Shapiro–Wilk test indicate a deviation from the normal distribution for some variables, and therefore, nonparametric tests were dedicated to them. The next step used r-Pearson's and rho-Spearman's correlation for investigation of the specific relationships between measured variables (Table 2).

For all the emotional deficits measured in the study, positive correlations were observed, but their strength varied. The strongest correlation was between alexithymia and emotional processing and explained ca. 49% of variation; the weakest one was between alexithymia and perceived stress as it only explained 7%.

In the next step of analysis, the results were compared to normalized data. We based this on English norms for alexithymia because there are no Polish norms for TAS-20 yet (cf. Association for Contextual Behavioral Science, https://contextualscience.org/TAS_Measure#).

As far as we know, alexithymia and emotional processing as possible predictors of bruxism have never been studied before. It has been suggested that bruxism is related to mood disorders and may also be associated with stable individual differences regarding the tendency to experience negative emotions [41]. We identified that 62% of patients were nonalexithymic, 15% of them in whom alexithymia was possible, and 23% of patients who revealed alexithymia. The difference in numbers is statistically significant (*χ*^2^ (2, *N* = 54) = 19.07; *p* < 00.01). The observed frequency of alexithymic patients is twice as high as indicated in the epidemiological data. Alexithymia is estimated at 10–13% in the general population. Accordingly, it is 12.8–17% in male groups and 25% in our study, and 8.2–10% in female groups and 21% in our study.

For emotional processing, Student's *t*-test was used for the group. The data were related to standardized data in the Polish population [46]. Tests showed that the patients had statistically significant lower deficits in emotional processing (*M* = 3.35; SD = 1.51) than the general population (*M* = 3.9; SD = 1.5) (*t*(52) = −2.6; *p* < 0.05). This suggests that patients with bruxism process emotions quite effectively.

In our study, the Stens scale was used for perceived stress [49]. This showed that ca. 81% of patients manifested a high level of stress and 19% with an average level (*χ*^2^ (1, *N* = 54) = 38.44; *p* < 0.001). Nobody showed a low level.

These data showed stable associations between bruxism and stressful life styles observed in other studies where bruxism has been reported to be correlated with stress [8, 35–39]. The findings of Abekura et al. [37] suggest that there is an association between sleep bruxism and psychological stress sensitivity. The case reported by van Selms et al. [39] corroborates the correlation between experienced stress and daytime tooth clenching. The significantly positive association of frequent bruxism with experiencing severe stress has also been proved by Ahlberg et al. [38]. However, the relationship between bruxism and job-related stress seems to be different. Studies of Japanese populations by Nakata et al. [35] concluded that bruxism is weakly associated with job-related stress in men but not in women.

The results of our study support the suggestions that bruxism may be a symptom of ongoing stress in normal work life [38] and is rather strongly associated with the general stress of everyday life. This, in turn, supports the thesis that the prevalence of bruxism depends on the development of civilization and modern lifestyles [22].

In our study, 37% of patients manifested a low state-anxiety level, 36% an average level, and 29% a high level, the differences being statistically insignificant. Similarly, 42% of patients manifested a low trait-anxiety level, 29% an average level, and 29% a high level. Most studies report the association of bruxism with anxiety and depression. As we did not investigate depression, we can relate our results only to anxiety. It is suggested that the association between anxiety and bruxism starts in childhood and persists into adulthood [53]. However, no association between bruxism and psychosocial factors in children younger than 5 years old has been found. A significant association between bruxism and stressful, anxious, and tense personality traits emerge in children older than 6 years and is present up to adolescence [54]. The results of our study do not support the association between bruxism and anxiety previously reported [4, 42–45, 55, 56].

On the basis of data in the specialist literature, which indicated that women suffer bruxism more often than men [15, 33, 60], intergroup comparisons were conducted which indirectly confirmed this according to the gender of the participants freely recruited into our study. There was no statistically significant difference between women and men in the main variables. There was only a difference in the avoidance of emotional processing. Men declared they used this strategy more often (*M* = 4.33; SD = 2.03) than women (*M* = 3; SD = 2.01) (*F* (1.51) = 4.67; *p* < 0.05).

Afterwards, for the further specification of female's and male's bruxism, we correlated the main variables for women and men separately. Nine correlations were possible, and all of them were identified in the female group but only 4 of them in the male group. It shows that women suffering from bruxism manifested more compact view of psychoemotional deficits then men. Next, we calculated the Fisher r-to-z transformation value to assess the significance of the difference between these two correlation coefficients (Table 3). A statistical tendency was only found for the correlation between alexithymia and emotional processing. The correlation in the female group was stronger than in the male group. There are positive correlations between all the variables in the female group, with a very strong correlation between alexithymia and emotional processing that explains ca. 58% of variation. The correlations in the male group are selective; the strongest one being between alexithymia and trait anxiety that explains ca. 56% of variation.

As most previous studies investigated self-reported bruxism, the strength of the present study is the dentist-assessed physical evidence of bruxism, with the additional use of the RDC-TMD questionnaire. The limitations that need to be addressed, however, include the criteria for this assessment. This problem is well known and debatable. Neither occlusal relationships nor the pain are believed to be definitive symptoms of bruxism. According to Lobbezoo and Naeije [16], occlusal interferences have no relevance to bruxism. Ommerborn et al. [14] evaluated 16 occlusal and functional parameters and found no statistically significant differences between sleep bruxers and controls. The assessment of tooth wear additionally failed to be a reliable diagnostic tool due to the high prevalence rate of tooth wear in nonbruxists [55–57]. Manfredini and Lobbezzo [17] and Paesani [6] stated that the use of pain for clinical diagnosis is also unreliable, because the relationship between bruxism and pain is controversial.

We find the age distribution within the clinical sample to be another strength of the study. Most of the participants were younger than 30 years old. This is convergent with the reported age dependence of bruxism occurrence in Hublin et al. [30]. Lavigne and Montplaisir [58] showed the linear decrease of bruxists from 13% among 18–29 years old to 3% among those aged 60 and older. Also, the proportion of females to males within the investigated group reflects the previously published results showing that the prevalence of bruxism is higher among females [12, 30, 56]. However, future research would benefit from more a numerous sample.

Despite a few limitations, we offer the first evidence that bruxism is not associated with emotional processing deficits; however, it is related to stress and alexithymia. Also, state anxiety and trait anxiety are important factors in bruxism.

The significance of psychosocial functioning is believed to be an essential part of the diagnostic process [59]. Because patients with anxiety and who experience stress seem to have a higher number of risk factors for bruxism [60], the profile of the data obtained suggests the possibility of preventing or minimizing the symptoms of bruxism with a properly constructed psychoprophylactic interaction.

## 4. Conclusions

Specific psychosocial functioning is a significant factor in explaining the determinants of awake bruxism. Psychosocial factors such as state anxiety and trait anxiety, stress, and alexithymia are as important as somatic causes in the occurrence and maintenance of bruxism. Our study revealed that most of the bruxers are nonalexithymic patients, but all of them revealed high or average level of stress. Further studies are needed to replicate our finding in larger samples and with more varied psychosocial determinants (e.g., age, generation, education, and employment). Moreover, longitudinal study may also provide valuable insights into the casual mechanisms behind the observed associations.

## Figures and Tables

**Table 1:** Descriptive statistics.

Variable	Min	Max	*M*	SD	*α*	Shapiro-Wilk test	
						*W*	*p*
Total alexithymia	25	71	48.59	12.59	0.85	0.96	0.105
Difficulty describing feelings	5	23	13.67	4.87	0.80	0.95	0.066
Difficulty identifying feelings	7	29	16.67	6.34	0.79	0.94	0.020
Externally oriented thinking	9	29	180.26	4.41	0.32	0.98	0.456
Total emotional processing	0	7.36	3.35	1.51	0.92	0.98	0.686
Suppression	0	7.8	3.38	2.08	0.89	0.97	0.313
Unprocessed	0	9.0	4.50	2.21	0.88	0.97	0.533
Controllability	0	6.4	3.26	1.59	0.68	0.97	0.356
Avoidance	0	6.4	3.41	1.69	0.64	0.95	0.043
Experience	0	7.80	2.19	1.67	0.80	0.93	0.012
Total perceived stress	14	31	22.92	3.85	0.87	0.98	0.782
Perceived helplessness	1	19	10.58	4.28	0.87	0.96	0.178
Perceived self-efficacy	5	16	10.31	2.74	0.69	0.97	0.330
State anxiety	22	58	38.05	9.24	0.92	0.92	0.005
Trait anxiety	25	66	41.98	9.64	0.91	0.95	0.037

**Table 2:** Correlation coefficient of investigated variables.

	1	2	3	4	5	6	7	8	9	10	11	12	13	14
(1) Total alexithymia	1													
(2) Difficulty describing feelings	0.86^*∗∗*^	1												
(3) Difficulty identifying feelings	0.89^*∗*^	0.71^*∗∗*^	1											
(4) Externally-oriented thinking	0.67^*∗∗*^	0.38^*∗∗*^	0.39^*∗∗*^	1										
(5) Total emotional processing	**0.7** ^*∗∗*^	0.65^*∗∗*^	0.66^*∗∗*^	0.37^*∗∗*^	1									
(6) Suppression	0.74^*∗∗*^	0.75^*∗∗*^	0.61^*∗∗*^	0.45^*∗∗*^	0.84^*∗∗*^	1								
(7) Unprocessed	0.37^*∗∗*^	0.37^*∗∗*^	0.37^*∗∗*^	0.12	0.76^*∗∗*^	0.43^*∗∗*^	1							
(8) Controllability	0.48^*∗∗*^	0.41^*∗∗*^	0.49^*∗∗*^	0.21	0.83^*∗∗*^	−0.6^*∗∗*^	0.64^*∗∗*^	1						
(9) Avoidance	0.6^*∗∗*^	0.51^*∗∗*^	0.6^*∗∗*^	0.31^*∗∗*^	0.84^*∗∗*^	0.65^*∗∗*^	0.49^*∗∗*^	0.62^*∗∗*^	1					
(10) Experience	0.69^*∗∗*^	0.57^*∗∗*^	0.67^*∗∗*^	0.41^*∗∗*^	0.83^*∗∗*^	0.76^*∗∗*^	00.31^*∗*^	0.63^*∗∗*^	0.72^*∗∗*^	1				
(11) Total perceived stress	**0.27** ^*∗∗∗*^	0.29^*∗*^	0.35^*∗*^	0.02	**0.46** ^*∗∗*^	0.29^*∗*^	0.46^*∗∗*^	0.48^*∗∗*^	0.32^*∗*^	0.32^*∗*^	1			
(12) Perceived helplessness	0.45^*∗∗*^	0.45^*∗∗*^	0.42^*∗∗*^	0.19	0.72^*∗∗*^	0.52^*∗∗*^	0.72^*∗∗*^	0.68^*∗∗*^	0.5^*∗∗*^	0.45^*∗∗*^	0.75^*∗∗*^	1		
(13) Perceived self-efficacy	−0.43^*∗∗*^	−0.42^*∗∗*^	−0.33^*∗*^	−0.33^*∗*^	−0.6^*∗∗*^	−0.51^*∗∗*^	−0.57^*∗∗*^	−0.5^*∗∗*^	−0.48^*∗∗*^	−0.41^*∗∗*^	−0.06	−0.63^*∗∗*^	1	
(14) State anxiety	**0.53** ^*∗∗*^	0.38^*∗∗*^	0.54^*∗∗*^	0.35^*∗*^	**0.6** ^*∗∗*^	0.44^*∗∗*^	0.52^*∗∗*^	0.54^*∗∗*^	0.45^*∗∗*^	0.45^*∗∗*^	**0.45** ^*∗∗*^	0.63^*∗∗*^	−0.53^*∗∗*^	1
(15) Trait anxiety	**0.64** ^*∗∗*^	0.54^*∗∗*^	0.65^*∗∗*^	0.42^*∗∗*^	**0.58** ^*∗∗*^	0.49^*∗∗*^	0.52^*∗∗*^	0.48^*∗∗*^	0.41^*∗∗*^	0.43^*∗∗*^	**0.52** ^*∗∗*^	0.68^*∗∗*^	−0.54^*∗∗*^	**0.67** ^*∗∗*^

^*∗*^ *p* < 0.05; ^*∗∗*^*p* < 0.01; ^*∗∗∗*^*p* < 0.52 (all two-tailed significance tests).

**Table 3:** Comparison of the correlations between women and men.

Reference	Women (*n* = 36)<	Men (*n* = 16)	*Z*	*p*
Alexithymia: emotional processing	*r* = 0.76; *p* < 0.001	*r* = 0.52; *p* < 0.05	1.29	<0.09
Alexithymia: perceived stress	*r* = 0.31; *p* < 0.064	*r* = 0.18; n.s.	—	—
Alexithymia: state anxiety	*r* = 0.62; *p* < 0.001	*r* = 0.24; n.s.	—	—
Alexithymia: trait anxiety	*r* = 0.68; *p* < 0.001	*r* = 0.75; *p* < 0.001	—0.44	Ns.
Emotional processing: perceived stress	*r* = 0.44; *p* < 0.01	*r* = 0.55; *p* < 0.05	—0.45	Ns.
Emotional processing: state anxiety	*r* = 0.73; *p* < 0.001	*r* = 0.27; n.s.	—	—
Emotional processing: trait anxiety	*r* = 0.73; *p* < 0.001	*r* = 0.54; *p* < 0.051	0.94	Ns.
Perceived stress: state anxiety	*r* = 0.43; *p* < 0.01	*r* = 0.36; n.s.	—	—
Perceived stress: trait anxiety	*r* = 0.61; *p* < 0.001	*r* = 0.3; n.s.	—	—

## Data Availability

All data are available on request.
